# Potential Pitfalls of IgG4 Immunohistochemical Staining on Lesional Tissue in Cutaneous Acantholytic Disorders

**DOI:** 10.3390/dermatopathology11040041

**Published:** 2024-12-19

**Authors:** Carla Stephan, Linglei Ma

**Affiliations:** 1 Department of Pathology and Laboratory Medicine, Weill Cornell Medical Center, NewYork-Presbyterian, New York, NY 10065, USA; qgh9002@nyp.org; 2 Department of Pathology, University of Virginia, Charlottesville, VA 22903, USA

**Keywords:** acantholytic disorders, IgG4, immunohistochemistry, acantholysis, Grover disease, bullous impetigo, immunobullous disorders

## Abstract

The diagnostic utility of immunohistochemistry on paraffin-embedded sections in bullous disorders is useful when frozen tissue is not available. In pemphigus vulgaris and pemphigus foliaceus, an intercellular lace-like staining pattern of IgG4 on lesional tissue by immunohistochemistry has been described, with a comparable sensitivity and specificity to direct immunofluorescence on perilesional tissue. This study aimed to evaluate the staining pattern of IgG4 in non-immunobullous disorders to highlight the potential pitfalls when using this stain. In this study, we conducted a retrospective review of our institution’s database of non-immunobullous disorders where immunohistochemistry of IgG4 was performed to rule out pemphigus. We identified 27 cases where IgG4 immunohistochemistry was performed and observed intercellular IgG4 staining in some cases of Grover disease, bullous impetigo, irritated dermal hypersensitivity reaction, acantholytic actinic keratosis, and graft versus host disease. Our results indicate that the interpretation of IgG4 staining by immunohistochemistry in cutaneous acantholytic disorders should be approached with caution. Confirmation on cryosections with direct immunofluorescence study results is important in these settings.

## 1. Introduction

Direct immunofluorescence (DIF) study remains the gold standard in the diagnosis of immunobullous diseases, with a high sensitivity and specificity. DIF studies require the utilization of frozen sections of perilesional, uninvolved skin samples. However, frozen tissue in Michel’s media for DIF may not always be available, especially when immunobullous diseases are not considered clinically. In addition, the referring laboratory may not be equipped to perform DIF. The diagnostic utility of IgG4 immunohistochemistry (IHC) on paraffin-embedded sections in immunobullous disorders has been shown to be useful [1,2]. In pemphigus vulgaris (PV) and pemphigus foliaceus (PF), an intercellular lace-like staining pattern of IgG4 has been described, with a sensitivity and specificity comparable to DIF [1,2]. IgG4 IHC is particularly valuable when DIF study is not available. However, only a few studies are in the literature on this topic. In our practice, we have encountered several non-pemphigus acantholytic conditions where IgG4 was performed to rule out immunobullous diseases based on histopathological findings. We found that some of these acantholytic lesions can demonstrate IgG4 intercellular staining in the epidermal keratinocytes as those observed in PV and PF. In this study, we aimed to investigate the potential pitfalls of using the IgG4 immunostain when diagnosing acantholytic disorders.

## 2. Materials and Methods

Under institutional IRB protocol (20-02021524), a retrospective search of one author’s (CS) institution’s database was conducted. A CoPath natural language search from the years of 2018 to 2023 was used to retrieve cases of acantholytic disorders where IgG4 IHC was utilized. The search terms “acantholysis”, “Hailey–Hailey”, “Grover”, “bullous impetigo” were used in conjunction with “IgG4”. The slides were retrieved and reviewed. We collected 27 cases of non-immune-mediated acantholytic dermatoses where IgG4 immunostaining was performed. In all cases, the biopsies were taken from the lesional tissue. The diagnoses of the included cases had been confirmed through clinical correlation, follow-ups, and subsequent biopsies. IgG4 immunohistochemical staining was performed on formalin-fixed paraffin-embedded (FFPE) tissues. Deparaffinization was performed on FFPE sections before antigen recovery was conducted. The antigens were detected using a 2-step immunohistochemistry procedure. Incubation with primary antibodies was performed, and then staining via the Vision BioSystems Define Kit (Norwell, MA, USA) was conducted. The IgG4 stain used was a mouse monoclonal antibody (MRQ-44) manufactured by Cell Marque (Rocklin, CA, USA). A pre-diluted formulation (Cat# 367M-18) was utilized. These cases were reviewed independently by two pathologists (CS and LM). The staining patterns for IgG4 were recorded. A continuous intercellular lace-like staining of the epidermal keratinocytes was scored as positive. A positive staining was further subcategorized into focal (<50%) or diffuse (>50%). The localization of IgG4 immunoreactivity to the upper half, lower half, or entire epidermis was also recorded separately. A homogenous cytoplasmic staining associated with dyskeratotic keratinocytes, debris, and exudates was considered nonspecific and was recorded as negative. Interobserver agreement on the grading between the two authors was reached for all cases.

## 3. Results

We identified a total of 27 cases of non-pemphigus, acantholytic conditions. These cases included Grover’s disease, Hailey–Hailey disease, bullous impetigo, acantholytic actinic keratosis, irritated dermal hypersensitivity reaction, eczematized chronic graft versus host disease, and impetiginized basal cell carcinoma. The results are summarized in Table 1. Of these 27 cases, DIFs were performed in 4 cases (2 cases of Grover disease and 2 cases of bullous impetigo) and were negative in all 4 cases. One case was originally diagnosed as bullous impetigo, but was later reclassified as IgA pemphigus upon a subsequent biopsy for DIF. This case was excluded from the study.

**Figure 1:**
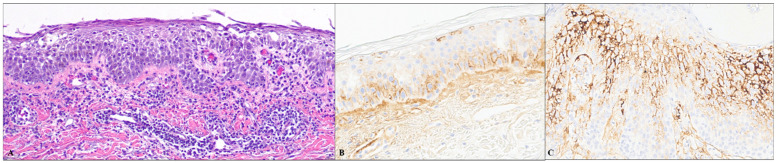
(**A**) H&E image of a skin biopsy of bullous impetigo (100×); (**B**,**C**) Immunohistochemical staining for IgG4 displaying focal intercellular staining in the lower half of the epidermis in one case ((**B**)—100×) and both upper and lower epidermis in another case ((**C**)—200×).

**Figure 2:**
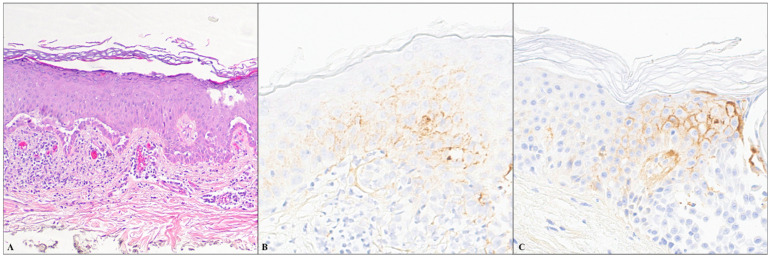
(**A**) H&E images of a skin biopsies of Grover disease (100×); (**B**,**C**) Immunohistochemical staining for IgG4 displaying focal intercellular staining in the lower half ((**B**)—200×) and upper half of the epidermis ((**C**)—200×).

**Figure 3:**
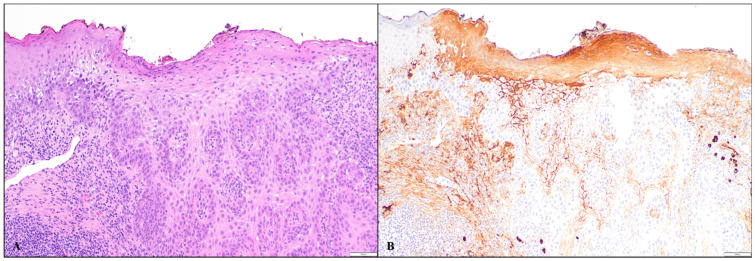
(**A**) H&E image of a skin biopsy of acantholytic actinic keratosis (100×); (**B**) Immunohistochemical staining for IgG4 displaying diffuse intercellular epidermal staining in both upper and lower half of the epidermis (100×).

Among 27 cases, 12 showed IgG4 intercellular staining, and 15 were negative for IgG4. The majority (11/12) had focal staining, while one case displayed diffuse reactivity. Three of four cases of bullous impetigo exhibited focal intercellular staining of IgG4 in the epidermal keratinocytes. Of these three cases, two exhibited focal staining in the entire epidermis, while one case showed staining in the lower half of the epidermis (Figure 1A–C). Three of thirteen cases of Grover’s disease displayed focal intercellular staining. Of these three cases, the IgG4 staining was confined to the lower half of the epidermis in one case, the upper half in the second case, and the entire epidermis in the third case (Figure 2A–C). Two cases of acantholytic actinic keratosis revealed intercellular IgG4 staining in both the lower and upper halves of the epidermis, with one case exhibiting diffuse staining and the other case showing focal staining (Figure 3A,B). All three cases of Hailey–Hailey disease were negative for IgG4. Three cases of irritated dermal hypersensitivity reaction (DHR), including two arthropod bite reactions and one drug-related hypersensitivity reaction, showed epidermal spongiosis/acantholysis raising concern for an acantholytic disorder, and therefore IgG4 stain were performed. One case of drug-related hypersensitivity reaction and one case of arthropod bite reaction displayed focal IgG4-reactivity in the lower half of the epidermis, while the second case of arthropod bite reaction was negative for IgG4. One case of traumatized basal cell carcinoma with superficial acantholysis showed focal full epidermal IgG4-positivity in the impetiginized area. One case of graft versus host disease displayed focal full epidermal IgG4 staining in the spongiotic area.

## 4. Discussion

There are four subclasses of IgG antibodies, IgG1–4, in the order of their discovery and serum concentration. IgG4 is a subclass of IgG that is involved in various skin conditions, including pemphigus vulgaris, pemphigus foliaceus, and bullous pemphigoid. Patients with pemphigus develop autoantibodies of the IgG4 class to desmoglein. In a study, circulating IgG4 was found in 62% of pemphigus vulgaris cases and was absent in the controls by indirect immunofluorescence staining, supporting the role of the IgG4 subclass in the pathogenesis of pemphigus [3]. IgG4 is the pathogenic autoantibody in PF and may also be important in other autoimmune diseases [4].

DIF performed on perilesional tissue remains the gold standard for the diagnosis of autoimmune bullous disorders [5]. Generally, DIF testing on frozen sections is performed with IgG, IgM, IgA, C3, and fibrinogen conjugates. In PV, an epidermal intercellular staining of IgG and/or C3 is observed by DIF. Recently, it has been shown that the addition of IgG4 to the DIF panel increases the diagnostic sensitivity, especially for cases with equivocal IgG and C3 staining. Like IgG, IgG4 shows cell-surface or linear deposition along the basement membrane zone in PV or bullous pemphigoid, respectively. However, IgG4 often has less non-specific background staining than IgG or C3 [6]. Another study found that modified DIF using a single fluorescein conjugate against IgG and IgG4 showed comparable results to the traditional DIF using separate fluorescein conjugates for IgG and IgG4. The authors suggest that the modified DIF can serve as a more cost-effective substitute for the traditional DIF [7].

The use of IgG4 IHC on paraffin sections for pemphigus was first introduced in 2012 [1]. In this study, 18 pemphigus cases and 36 control specimens were analyzed. IgG4 staining with an intercellular lace-like pattern localized to the suprabasilar keratinocytes was only found on the lesional biopsies (with acantholysis/preacantholysis), whereas the perilesional biopsies were negative. It was found that IgG4 immunostaining for the diagnosis of pemphigus had an overall sensitivity of 72.2% and specificity of 97.2%. This intercellular lace-like staining pattern is similar to those seen in DIF studies for PV. In 2019, another group showed that IgG4 IHC had a higher sensitivity in specimens with PF (83.3%) as opposed to PV (72.4%) [8]. A more recent study reported a sensitivity of 85.7% and specificity of 96.3% for IgG4 IHC for pemphigus [9].

In the original 2012 series, 4 normal skins and 32 non-pemphigus bullous diseases were used as controls. Only a single case of bullous pemphigoid showed intercellular staining in the suprabasilar keratinocytes, while all other controls were negative. One case of bullous impetigo demonstrated focal staining above the area of blistering [1]. It was speculated that this nonspecific staining may have resulted from exudate permeating into the spongiotic cellular junction. In a 2017 study, the authors reported IgG4 intercellular positivity in the basilar keratinocytes among 8 of 10 bullous pemphigoid cases [10]. More recently, another group also observed IgG4 positivity at the basal layer in 1 of 25 bullous pemphigoid cases [9]. Interestingly, in the 2019 study, the authors reported positive staining for IgG4 in 6 out of 35 control specimens (4 normal skins and 31 basal cell carcinoma) [4]. However, the authors did not specify the details of these six specimens with positive IgG4 staining in their report.

In this study, we found that focal and occasionally diffuse intercellular IgG4 staining can be seen in the epidermal keratinocytes in some non-pemphigus acantholytic conditions. Of the 27 cases, 12 cases (44%) were found to have an IgG4 intercellular lace-like staining pattern similar to that observed in pemphigus. While the majority showed focal staining, one case of acantholytic actinic keratosis demonstrated diffuse IgG4 positivity, posing potential diagnostic challenges. This case of acantholytic actinic keratosis showed evidence of excoriation, spongiosis, and impetiginization. Despite the presence of IgG4 staining, the diagnosis of actinic keratosis fits best given the solitary nature of the lesion and additional clinical information provided. The patient underwent treatment with photodynamic therapy for the lesion.

Some of the encountered IgG4-positive cases, including both cases of actinic keratosis, all three cases of bullous impetigo, one GVHD, and one basal cell carcinoma, had focal excoriation and spongiosis in addition to acantholysis. All three cases of dermal hypersensitivity reactions had focal spongiosis, papillary dermal edema, or early subepidermal bulla, which raised concern for bullous pemphigoid. Of all the positive cases, the localization of the IgG4 staining (upper, lower, or entire epidermis) varied in each case. The positively stained areas are often aligned with the areas of acantholysis and spongiosis.

**Figure 4:**
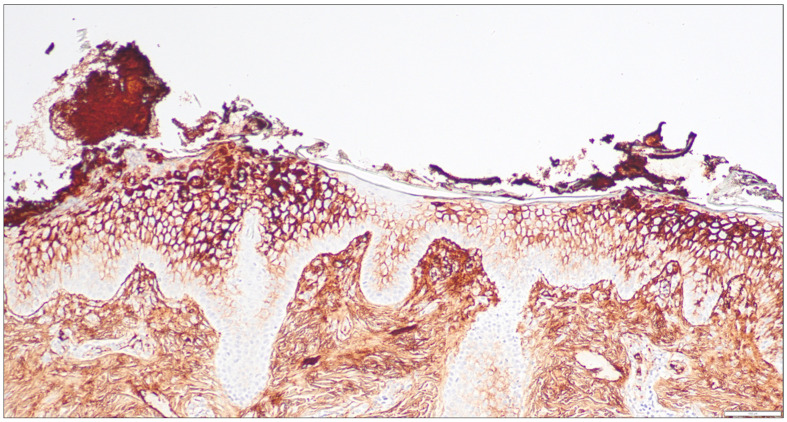
Immunohistochemical staining for IgG4 in a skin biopsy of IgA pemphigus (100×).

One case of IgA pemphigus was encountered in our study. The initial biopsy suggested bullous impetigo. An IgG4 immunostain was performed and showed diffuse positivity in the upper epidermis sparing the basal layer (Figure 4). No DIF was performed at the time. However, the patient’s rash progressed, and a subsequent biopsy with DIF confirmed the diagnosis of IgA pemphigus. The IgG4 IHC positivity seen in the initial biopsy is interesting. It is known that IgA pemphigus may show intercellular IgG deposits by DIF [11]. This may explain the presence of intercellular IgG4 by IHC in this case [1,8,9].

Post-pemphigus acanthoma is a verrucous lesion at the site of a previous blister in individuals with apparent clinical remission of pemphigus. Interestingly, there is evidence of post-pemphigus acanthoma showing intercellular fluorescence by DIF, suggestive of an active disease [12]. IgG4 IHC staining on this entity has not been described and would be worth exploring in the future.

The mechanism by which intercellular IgG4 staining is seen in non-immunobullous acantholytic disorders is unknown. The loss of cell adhesion due to acantholysis and/or spongiosis may result in the uncovering of adhesion molecules on the transmembrane of keratinocytes. The intercellular IgG4 binding without auto-antibodies in the non-pemphigus acantholytic conditions is likely a result of nonspecific cross-reactivity. Further studies with more cases are needed to assess the nature of IgG4 staining in various non-immunobullous diseases.

In conclusion, although immunohistochemistry of IgG4 can be a valuable tool in the diagnosis of pemphigus, a positive IgG4 staining should be interpreted with caution as some non-immunobullous acantholytic disorders may display focal intercellular IgG4 staining. Clinical information, subsequent DIF study results, mosaic-based indirect immunofluorescence, or multiplex ELISA testing should be judged carefully to avoid potential diagnostic pitfalls.

## Figures and Tables

**Table 1:** Summary of IgG4 staining results in non-immunobullous acantholytic disorders.

	IgG4 Staining Pattern		
	Negative	Focal Staining	Diffuse Staining
Grover Disease (*N* = 13)	10	3	0
Bullous Impetigo (*N* = 4)	1	3	0
Hailey–Hailey Disease (*N* = 3)	3	0	0
Graft Versus Host Disease (*N* = 1)	0	1	0
Actinic Keratosis (*N* = 2)	0	1	1
Dermal hypersensitivity reaction (*N* = 3)	1	2	0
Basal cell carcinoma (*N* = 1)	0	1	0
Total cases (*N* = 27)	15	11	1

## Data Availability

The original contributions presented in this study are included in the article. Further inquiries can be directed to the corresponding author.
